# Emotional responses to conspecific distress calls are modulated by affiliation in cockatiels (*Nymphicus hollandicus*)

**DOI:** 10.1371/journal.pone.0205314

**Published:** 2018-10-09

**Authors:** Agatha Liévin-Bazin, Maxime Pineaux, Olivier Clerc, Manfred Gahr, Auguste M. P. von Bayern, Dalila Bovet

**Affiliations:** 1 Laboratoire Éthologie Cognition Développement, EA, Université Paris Nanterre, Nanterre, France; 2 Laboratoire Évolution et Diversité Biologique, UMR 5174, Université Toulouse III Paul Sabatier, Toulouse, France; 3 Department of Behavioural Ecology and Evolutionary Genetics, Max Planck Institute for Ornithology, Seewiesen, Germany; SUNY Polytechnic Institute, UNITED STATES

## Abstract

Vocal communication is used across the animal kingdom to transfer information from emitters to receivers, such as size, sex, age, dominance status or even emotional states. The transmission of an emotional state from one individual to another is called “emotional contagion” and is classified as the first level of empathy. Emotional contagion is thought to be stronger between familiar individuals. While affiliation represents a stronger relation between individuals than mere familiarity, it remains understudied whether affiliation modulates emotional reactions as well. Using cockatiels (*Nymphicus hollandicus)*, we played back three types of audio stimuli to individual birds: a partner’s distress call (emitted when birds are caught or forcibly restrained), a non-partner’s distress call, and a control sound (white noise). The calls were recorded from familiar birds with either low (non–partners) or high levels of affiliation (partners). The subjects’ response was scored using four behavioural parameters: the time spent near the loudspeaker, the amount of movements, the number of calls emitted, and the position of the crest. Across all variables, birds were more attentive and active when confronted to distress calls compared to control sounds, particularly when the distress call was emitted from a partner rather than a non-partner. These results raise the possibility that distress calls do not only function as a stimulus-triggering automatic reaction in cockatiels but also transmit emotions. Moreover, affiliation enhanced emotional reactions to conspecific distress calls. Our data provides first insights into the mechanisms of emotional contagion in parrots.

## Introduction

Empathy, the ability to recognize and share the emotions of other individuals, has been described as a complex ability that can be split into three different levels of increasing complexity [1]. The lowest level, and less cognitively demanding form of empathy, is emotional contagion. It can be described as the triggering of a similar emotional response in both an emitter and a receiver [1, 2]. The transmission of emotional states from one individual to another may manifest in altered sensory, motor, and physiological states of others (i.e. increase in cortisol level, heart rate and locomotion) [3]. Emotional contagion is closely linked to automatic mimicry, the tendency to imitate and synchronize with the movements of others [4].

Acoustic communication can be a useful medium to investigate emotional contagion. Vocalizations encode a broad range of information about the emitter such as identity, age, weight [5], sex, kinship [6], dominance status [7] or emotional states[8–10]. Perceiving information about the emitter’s emotions can potentially induce the same emotional state in a receiver or may simply increase its emotional arousal [11]. Certain types of calls such as alarm or distress calls might have intense emotional salience and elicit interspecific fear and flight responses in receivers [12]. Distress calls are very specific loud calls emitted when an animal is in a situation of extreme distress, such as when caught by a predator [13]. They can serve different functions, like warning others about the presence of a predator, calling for help and distracting or mobbing predators [13–15]. In birds, distress calls of different species show parallels in acoustic structure and often elicit interspecific responses [16]. However, birds respond significantly stronger to conspecifics’ distress calls than to those of more distantly related species [17]. The majority of experiments investigating the reaction to conspecifics’ calls use mammals, such as goats[9, 10], horses [18], pigs [19], dogs [20], rodents [21], but also lizards have been tested [22]. In birds, most of the studies have focused on structural [23] and functional aspects [13, 15] of distress calls, however, they did not take into account the social bonds between emitters and receivers of these calls.

The magnitude of an individual’s reaction to others’ behaviours might additionally depend on within-species parameters, such as familiarity (i.e. individuals know each other and can be genetically related or unrelated), or affiliation (i.e. individuals choose to interact preferentially with certain conspecifics). Familiarity can modulate an emotional reaction to conspecific behaviour although there is mixed empirical support for this (e.g. in horses [18], dogs [20], and mice [24]). While the role of familiarity in emotional contagion has been investigated, few studies have looked at the effect of affiliation, which describes an even stronger social bond than familiarity. Affiliative relationships are commonly described as high quality social bonds between individuals that are not necessarily genetically related [25]. Individuals sharing an affiliative relationship exchange socio-positive behaviours (e.g. allopreening/allogrooming or allofeeding) and spend time in close spatial proximity [25–27]. Affiliation has been shown to have an impact on emotional contagion in primates. In bonobos (*Pan paniscus)* and humans, yawn contagion is higher between individuals sharing affiliative relationships than between non-affiliated individuals [28]. Up to now, to our knowledge, no study has tested the effect of affiliation on emotional contagion in other taxa than mammals.

Several bird species like rooks (*Corvus frugilegus*) [29, 30], jackdaws (*Coloeus monedula*) [31], ravens (*Corvus corax*) [32] and parrots [33] live in individualised societies comparable in complexity to those of primates [34, 35] and show similar affiliative association patterns. In birds, affiliative behaviours have been observed between sexual partners [36, 37], siblings [38], and unrelated individuals of the same sex [30]. Affiliative behaviours with mates or non-sexual partners can provide better access to food [39], facilitate coalition formation through cooperation [40] or enable consolation following stressful situations [41]. A few studies have explored emotional arousal and contagion between familiar conspecifics in birds, like geese [42–44], psittacids [45–48] and corvids [49], all of which form long-term monogamous pair-bonds [50]. A study found that graylag geese (*Anser anser*) exhibited increased heart rate when they observed the pair partner or a family member engaging in agonistic interactions, while no changes in heart rate occurred when they witnessed fighting between non-affiliated individuals [51].

Psittacids are interesting candidates to investigate responses to conspecific calls, since it has already been demonstrated that they possess individual vocal signatures [52, 53], and are able to recognize others by their voice [54–56]. In the social domain, parrots are characterised by their complex social life [57, 58], their longevity, their long-term monogamous pair-bonds [59, 60] and their ability to cooperate [61] and act prosocially [62]. In the present study, we chose to study the response of cockatiels (*Nymphicus hollandicus*) to distress calls of conspecific affiliative partners and non-partners. Cockatiels are small Australian parakeets that belong to the cockatoo family. They are a highly social species, which form long-term monogamous pair-bonds and live in large fission-fusion flocks [63]. Like other members of the cockatoo family [64], they exhibit a rich vocal repertoire and seem to be capable of individual vocal recognition.

To test whether the affiliation between individuals would be reflected in the strength of the behavioural response when confronted with distress calls, we exposed individual birds to two types of distress calls: the distress calls of a partner, a bird with which the subject maintained a strong affiliative bond, and the distress calls of a non-partner, a familiar bird housed in the same aviary without particular relations to the subject. Given the complex affiliative relationships found in these bird species, we predict that affiliation modulates emotional responses to conspecifics’ behaviour and consequently, that birds would react more intensively to partner’s distress calls than to non-partners calls. In order to rule out that the birds’ reaction was simply driven by a reaction to the sound rather than the emotional valence of the stimuli, a control sound (white noise) was additionally included. We recorded and analysed the behavioural responses of the birds before, during and after playback of the stimuli. We predicted that if the birds recognize the emotional content of the stimuli, they would show stronger responses to the distress calls (i.e. alertness and stress behaviours) than to the artificial control sound. Moreover, if the birds are able to individually recognize the distress calls from their partners and if emotional contagion is affected by affiliation, we predicted that the birds would show the strongest response to distress calls produced by their partners while responding to those of other group members to a lesser degree.

## Materials and methods

### Ethical statement

All the birds were housed in standard conditions approved by the French National authorities. This study complies with French and European legislation for animal care and was approved by the Ethical Committee for Animal Experimentation Charles Darwin (authorization number 2015031616168767 v6 (APAFIS#344)). Birds were never food deprived during experiments. The stressful event created to obtain distress calls from the birds was kept very short (approximately 1 minute) and was authorized by the authorities.

### Subjects and housing conditions

A flock of ten young cockatiels (6 females and 4 males) aged between 1 and 2 years were used in this study. All birds hatched in captivity and arrived at the Laboratoire Éthologie Cognition Développement in Paris-Nanterre University at ca. three months of age. The birds were housed together in an indoor aviary (296 cm x 257 cm x 257cm) with three stainless steel tables (155 cm x 55 cm x 84 cm) covered with Kraft paper, two large perch structures (2 meters long), two triangular bird swings and multiple toys hung from the roof. The room was kept on an automated light-dark cycle (time on: 0800, time off: 1900) with UV daylight tubes with a light spectrum especially adapted for birds (Arcadia Bird lamp T8). The temperature was maintained at 25 C°. Extrudes granules (Nutribird G14), anise sand, fresh fruits, vegetables, as well as bathing and drinking water were provided *ad libitum*.

Birds arrived in the lab in two phases: a pair of siblings (Nephtys and Seth) and the five other unrelated birds (Wala, Sita, Callisto, Viviane and Hermes) arrived in the laboratory in October 2013, while three further unrelated birds (Odin, Skadi and Loki) were acquired one year later, and were introduced to the common aviary in February 2015. Given the acquisition of additional birds at a later point in time, the study was performed in two parts: the first one was conducted with seven birds in December 2014 and the second one with the last three birds in July 2015.

### General experimental procedure

Each subject was tested with three different stimuli on three separate days: 1) artificial white noise, 2) distress calls from a partner (affiliated individual) and 3) distress calls from a non-partner (sharing no affiliative bond). The conditions and the order of the stimulus presentation were semi-randomised and counterbalanced across the subjects (see S1 Table). The experimental phase was approximately 30 minutes per bird. In order to minimize the separation stress, each day of testing was separated by 4 to 5 days without testing. The first seven birds were tested on the 26^th^ of November, 1^st^ of December and 5^th^ of December 2014, while the three last birds (Loki, Skadi and Odin) were tested on the 22^nd^, 27^th^ and 31^st^ of July 2015. Birds were tested during the day between 10 am and 5 pm. Birds were tested alone, one at a time, in a sound-proof chamber illuminated with white light to eliminate the possibility of being influenced by the presence of other birds in the room. Each bird was tested with distress calls emitted from individuals of the same sex and age. Because of the bias in sex ratio between subgroups (only one female, Nephtys, in the “siblings” subgroup and only one male, Hermes, in the first unrelated subgroup), calls of these individuals were used several times as non-partners stimuli. Behavioural responses of subjects were recorded with a webcam during the entire 30-minute duration of the experiment.

### Assessment of affiliative relationships

All birds were group-housed in the same aviary and thus familiar with each other. In order to create experimental dyads of partners and non-partners for each bird, we determined strong and weak affiliative relationship of each cockatiel in the group. Affiliative relationships were assessed via affiliation indexes based on socio-positive behaviours. In order to monitor the affiliative interactions within the group, videos of twenty-minutes were recorded at three different periods of time: 1) 6 videos in May 2014, six months before the first part of testing, in order to assess the general dynamics of the group; 2) 21 videos in December 2014 and January 2015, following the first part of testing, in order to validate the choice of the experimental dyads, and 3) 18 videos in April 2015, before the second part of testing, in order to establish the experimental dyads for the last three birds. Behavioural observations were recorded using a camera (Sony Handycam HDR-CX410) fixed on a tripod (Vanguard Mak 203) and coded based on the “all occurrences and continuous sampling method” [65]. Videos were analysed using the software VLC media player, version .1.5.

For each dyad, we constructed an index of affiliation following Silk et al’s procedure (2006) [66] which was adapted to birds by Boucherie et al (2016) [30]. We used the first set of observations recorded in May 2014 to create partner and non-partner dyads as presented in Table 1. However, the second set of observations done in December 2014 for another experiment on food-sharing [67] revealed that the affiliative relationships changed between the first and second sessions of observation. We observed that some affiliations, especially between males, observed in the first session of videos no longer existed in the second session for the seven birds. Consequently, we chose to use the data collected during the last two sessions (i.e. closest in time to the experimental periods) to create the index of affiliation even though relations had slightly evolved. These (small) differences in affiliation between the first and second sessions explain why not always the highest and lowest indexes of affiliation were used to form the dyads. The following behaviours were coded and included in the calculation: the time (in seconds) spent in spatial proximity to another bird (PROX, i.e. distance between two birds was so small that they could touch each other), and affiliative behaviours between birds (i.e. SOL: frequency of solicitation of allopreening; ALO: frequency of allopreening). Allopreening is considered the equivalent to allogrooming in primates. It was coded when a donor preened the receiver’s head or back and stopped with the donor lifting its head. A solicitation for allopreening is an easily recognized ritualized posture where one individual bows its neck presenting it to its partner. Solicitation was scored when the bird stopped requesting allopreening by lifting its head.

**Table 1:** Characteristics of the subjects and index of affiliation shared between partners and non-partners.

		Emitters									
		Callisto (F)	Hermes (M)	Nephtys* (F)	Seth* (M)	Sita (F)	Viviane (F)	Wala (F)	Loki (M)	Odin (M)	Skadi (F)
**Subjects**	**Callisto (F)**	/	0.28	*0*.*42*	0	**119.75**	0	457.10	NA	NA	NA
	**Hermes (M)**	0.28	/	*4*.*71*	1.80	115.50	**648.66**	29.21	NA	NA	NA
	Nephtys* (F)	0.42	*4*.*71*	/	**930.74**	0	4.15	1.52	NA	NA	NA
	Seth* (M)	0	1.80	**930.74**	/	5.95	2.08	*0*.*42*	NA	NA	NA
	**Sita (F)**	119.75	115.50	*0*	5.95	/	99.22	**231.05**	NA	NA	NA
	**Viviane (F)**	0	**648.66**	4.15	*2*.*08*	99.22	/	8.44	NA	NA	NA
	**Wala (F)**	**457.10**	29.21	*1*.*52*	0.42	231.05	8.44	/	NA	NA	NA
	**Loki (M)**	0	0	0	0.37	*0*	**48.92**	19.10	/	3.43	35.29
	**Odin (M)**	*2*.*29*	0	0.41	1.19	0	5.67	28.89	3.43	/	**103.13**
	**Skadi (F)**	3.93	0	0	*0*	53.06	0	6.58	35.29	**103.13**	/

The sex of birds (F: Female; M: Male) and affiliative indexes of all possible dyads in partners and non-partners duos are shown. Stars * indicate that birds (Nephtys and Seth) are siblings. The matrix needs to be read as follows: subjects’ names are written on the left side of the table and emitters’ names are written on top of the table. The dark grey cells who run diagonally indicate that birds could never hear their own calls. Each horizontal line indicates the affiliation shared between the subject and every other bird. For example, Viviane shares a score of 0 with Callisto, 648.66 with Hermes, 4.15 with Nephtys, 2.08 with Seth, 99.22 with Sita and 8.44 with Wala. No affiliation score was calculated between Viviane, Loki, Skadi and Odin since they hadn't arrived in the lab when Viviane was tested. Each subject heard the calls of an affiliate partner (high indexes in bold underlined numbers) and calls of a non-partner (low indexes in italics underlined numbers). Partner and non-partner for the same subject were same-sex individuals to avoid any bias. Loki, Odin and Skadi arrived after the first seven birds had been tested and could not be used as partners or non-partners at this time (as indicated by the mention “NA” and the light grey cells in the table, see text for details).

We calculated the affiliation index as follow:

$$S_{hv}=\frac{(({PROX}_{hv}/PROX)+({ALO}_{hv}/ALO)+({SOL}_{hv}/SOL))\;x\;100}{3}$$

The index of affiliation (S) was calculated separately for each dyad. In this example, it is calculated for the birds named ‘h’ and ‘v’ (S_*hv*_). PROX_*hv*_ (frequency of spatial proximity within dyad *hv*), ALO_*hv*_ (frequency of allopreening within dyad *hv*), SOL_*hv*_ (frequency of soliciting allopreening within dyad *hv*) are all separately divided by the respective overall frequency of occurrences of the specific behaviour across all possible dyads. The denominator is fixed and refers to the number of included variables. The value of the index of affiliation increases with the strength of a relationship.

Partners for the playback phase were selected based on high indexes (422.18 on average; minimum: 48.92; maximum: 930.74), while non-partners were chosen based on low indexes of affiliation (1.66 on average, minimum: 0; maximum: 4.71, see Table 1). The difference in the indexes of affiliation between partner and non-partner was at least 48.92.

### Recording procedure

**Fig 1:**
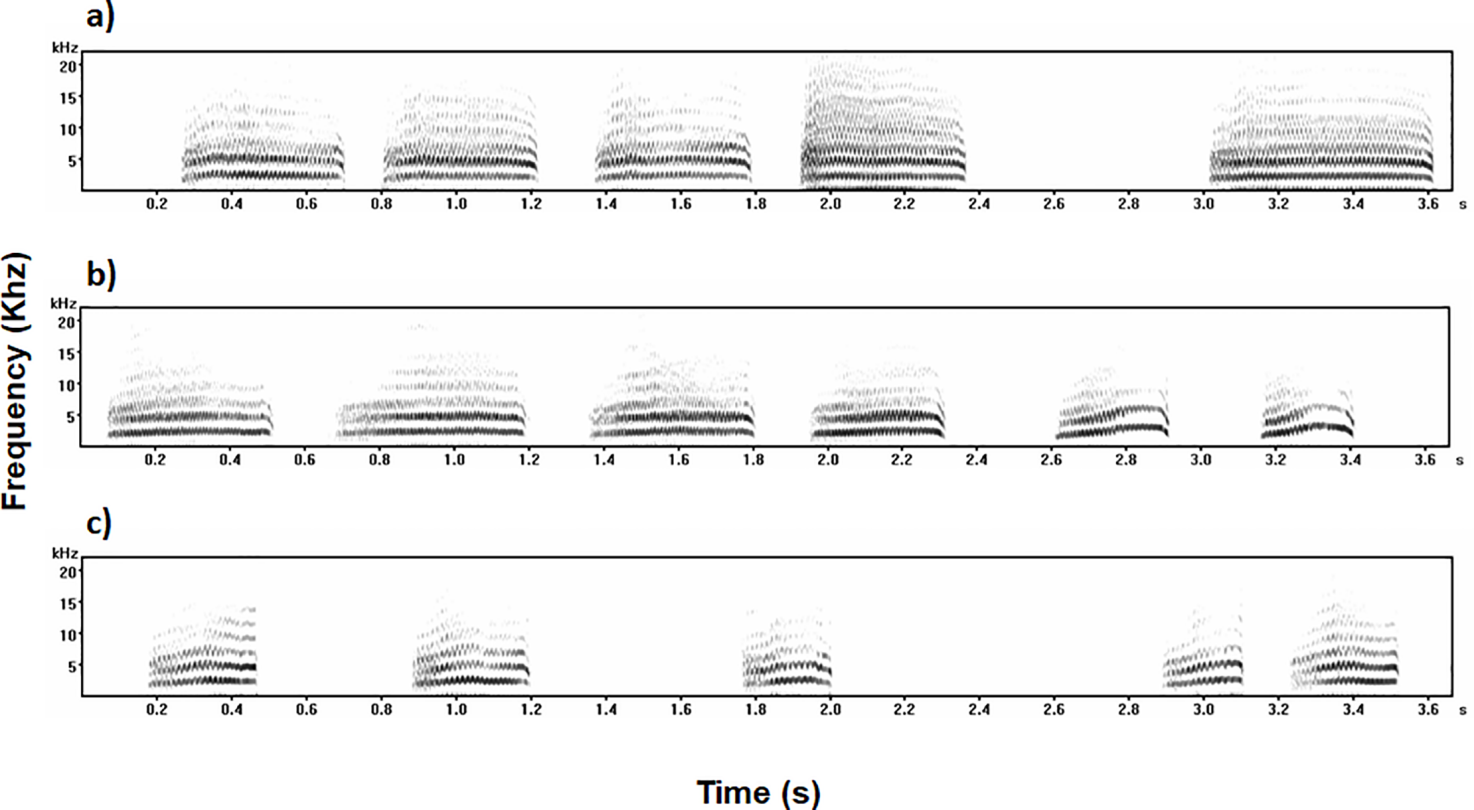
**Spectrogram displaying the first calls from (a) Wala, (b) Sita and (c) Hermes extracted from the distress call sequences used in the experiment**. Each playback stimulus consisted of 10 different calls from the same bird, which were repeated 3 or 4 times to obtain 30 seconds of playback for each emitter.

We created playback sequences of distress calls from each bird. These calls were obtained from recordings of a stressful event. For this purpose the bird was individually isolated in a cage (41 cm x 24 cm x 29.5 cm) placed in a sound proof chamber away from the aviary. An unfamiliar experimenter (to avoid fear reactions towards their usual experimenter), then inserted his hand in the cage for one minute, wearing a large leather glove. The same leather gloves were used when the birds were handled for medical care and were therefore associated by the birds with being seized. Consequently, birds started to emit alarm calls (i.e. in context of physical restraint) when they saw the glove. These distress calls are characterized by repeated harmonic harsh high-pitched calls (see Fig 1 for an exemplary spectrogram).

Calls were recorded for one minute per bird. The vocalizations were recorded in WAV format with a Sennheiser microphone (MD21U) set up in its stand (Sennheiser MZT 100) and a Marantz recorder (Marantz PMD 670; sampling rate: 44100 Hz; accuracy: 16 bits, mono). Each bird was released back into the aviary immediately after the recording session. One bird never emitted distress calls (Loki) and consequently he could only be tested as subject. Two recording sessions were conducted, one in October 2014 with seven birds, and another one in June 2015 with the remaining three birds.

### Preparation of the playback stimuli

Two types of stimuli were created: distress calls and artificial white noise. Both stimuli were computer-edited using Avisoft SASLab Pro, version 5.0.14 (Raimund Specht, Berlin, Germany).

In order to create comparable distress call stimuli from each individual, we selected 10 different calls from each bird per stimulus and individually normalized them to 75% with an automatic feature in Avisoft SASLab Pro software, which adjusts the intensity of the different calls. Only calls that were distinctly audible, with no sound saturation or environmental noise (like the sound of birds’ feet on the cage floor) were used to create the playback stimuli. For gaining playback stimuli comparable in intensity, we recorded all calls on the same day and later clipped them from the original recordings. When possible, we kept consecutive suitable calls (with no saturation or background noises) in the same order with the original silent intervals between them. The goal was to keep the final playback sequence as close to the original distress calls as possible. If we could not obtain 10 calls in a natural sequence, we added calls clipped out randomly from the recordings and placed short silent intervals of different durations (usually less than 1 second) in between. In the end, we were able to obtain 10 calls from 9 out of 10 birds.

In order to get a final duration of 30 seconds per playback stimulus, we repeated the 10 calls several times. We usually had 4 repetitions of 10 calls per 30 seconds of playback stimulus (with the exception of Seth and Viviane who emitted very long calls and consequently only 3 repetitions of 10 calls per 30 seconds were played back for these birds). The segmentation of each call was achieved with outline syllables parameters on Sound Analysis Pro 2011 software (Frequency range: 22050 Hz, FFT data window: 10 ms, Advance window: 1.5 ms, contour threshold: 10). The final single distress calls exhibited the following characteristics (±SE): mean duration of 502 ± 61 ms (range: 296–869 ms, n = 9); mean fundamental frequency of 2135 ± 114 Hz (range = 1677–2543 ms, n = 9), mean frequency of 3478 ± 181 Hz (range: 2874–4428 ms, n = 9).

For the broad-band white noise playback stimulus, a continuous audio stimulation lasting 30 seconds (no silence parts inserted) was automatically generated with a feature in AvisoftSASLab Pro software (0–22050 Hz). We chose to present white noise as control stimulus, as it can trigger attention, while it is free of any emotional value [19]. Each stimulus was played back at the same amplitude, with a maximum noise level of 91 dB (measured with a Ro-Line SPL meter, R0-1350, using ‘A’ weighting at the typical position of the test bird, 15 cm from the loudspeaker), which reflects natural amplitude levels.

### Experimental procedure

**Fig 2:**
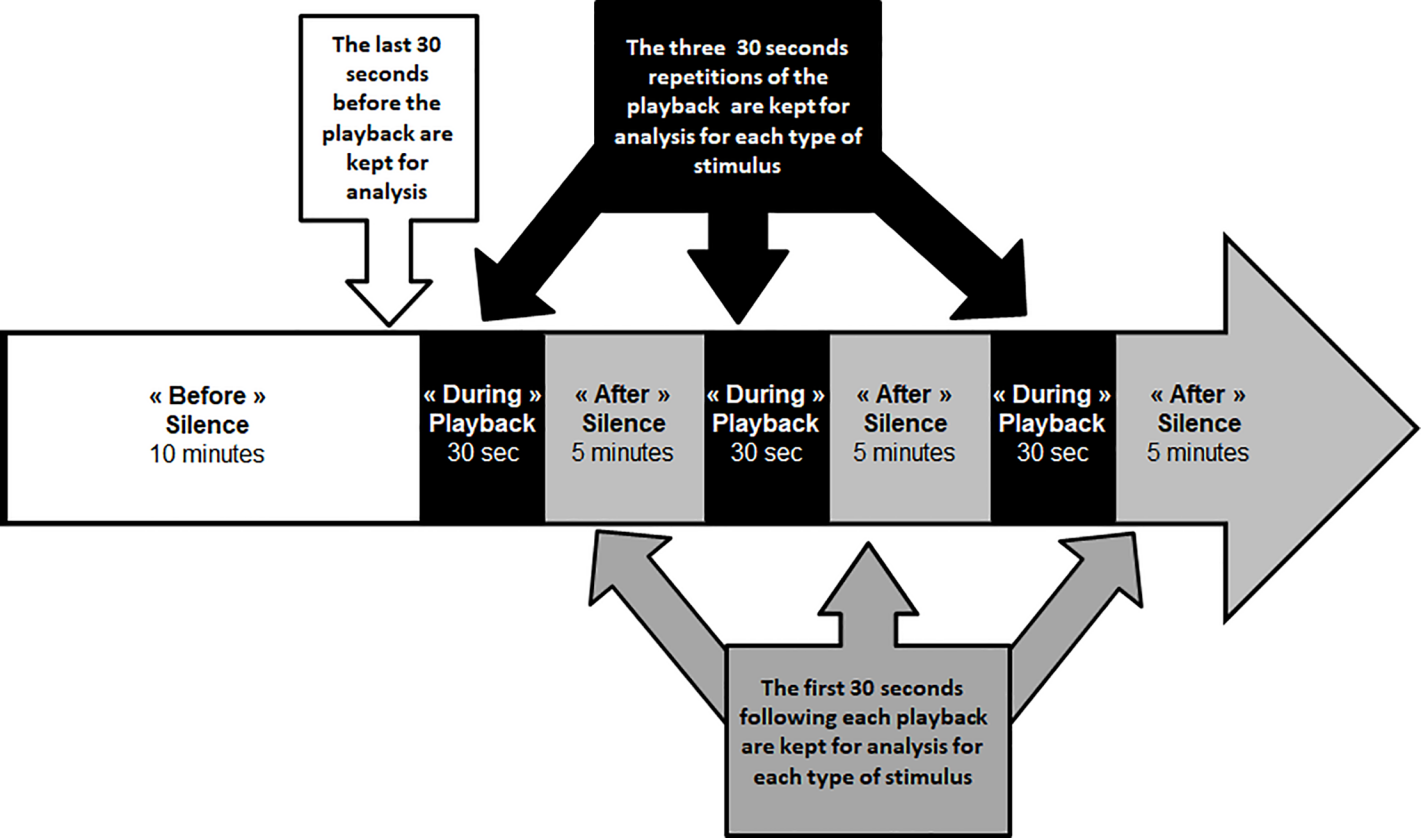
Schematic representation of the playback sequence. Three distinctive phases are represented: the 10 minutes silent phase before the playback stimulus (“Before” in white), the three 30 seconds repetitions of the playback stimulus (“During” in black) and the 5 minutes silent phases following each playback stimulus (“After” in grey). The arrows show the exact parts kept for analysis: the last 30 seconds of the “Before” phase, each repetition of 30 seconds of each stimulus in the “During” phase and the first 30 seconds of each “After” phase.

Each test comprised three distinctive phases: “before”, “during” and “after” the playback stimulus (Fig 2). It began with a phase of 10 min. silence referred to as “before”, which preceded the first playback stimulus. This silence phase was included to allow the bird to calm down after being removed from the aviary and habituate to the cage in the sound proof chamber. Then, the ‘during’ phase began, in which the playback stimuli were presented. Three repetitions of the same stimulus (either distress call or white noise) of 30 sec. were played back, with a 5 min. interval of silence in between the different playback stimuli. These silence phases of 5 min. following each playback stimulus, were called “after” and occurred three times in total per test.

**Fig 3:**
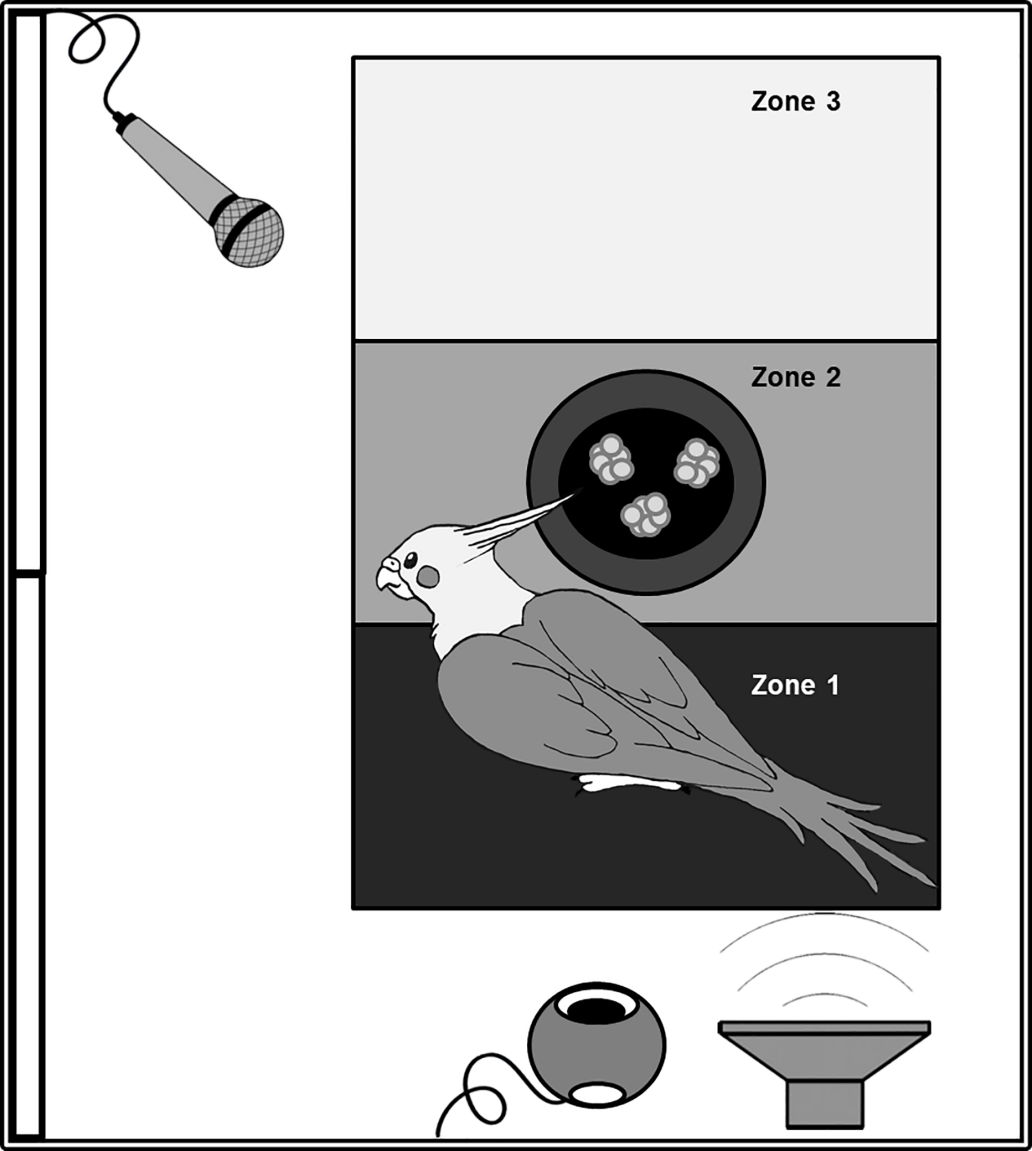
Experimental apparatus (Top view). The bird was placed inside a sound-proof chamber in a cage separated in three distinctive zones (zone 1 near the loudspeaker, zone 2 with a cup filled with millet, and zone 3 the furthest from the loudspeaker). The entrance of the sound-proof chamber was on the left via a two-way casement window. A webcam and a loudspeaker were positioned near the cage and a microphone recorded the subject’s calls.

The subject was caught and placed into a transport cage (41 cm x 24 cm x 29.5 cm). The cage was then moved in the experimental room and was positioned in a sound-proof chamber (69 cm x 49.5 cm x 49.5 cm). The opening of the sound-proof chamber consisted of a two-way casement window (Fig 3). On the right end, the sound-proof chamber was put near a wall. The experimenter left the room after closing the window of the sound-proof chamber and starting the broadcast of the stimulus. Consequently, birds were left alone during the entire experiment and could not see anything outside of the sound-proof chamber. The surroundings of the cage consisted of a microphone, the loudspeaker and the webcam, hung on the ceiling of the sound-proof chamber.

A cup filled with 10 grams of millet was placed in the middle of the cage. Three lines were drawn on a piece of kraft paper inserted in the bottom of the cage to create three distinctive zones (13.5 cm long for each zone) (Fig 3.). The first zone was closest to the loudspeaker, the second zone was the middle one, into which the cup with the 10 grams of millet was placed, and the third one was the most distant zone from the loudspeaker.

The behaviour of the birds was recorded using a webcam (Logitech HD Pro C920) connected to a computer (HP Pavillon dv6000). A Sennheiser microphone (MD21U) with its stand (Sennheiser MZT 100) was used to record calls emitted by the subject on a Marantz PMD 670 recorder. We played back the stimuli from a 60 Watts Mini Elipson Horus loudspeaker (frequency response: 80 Hz—20 kHz) placed in the back of the sound proof chamber at 15 cm distance from the cage. The loudspeaker was connected to a stereo amplifier Pioneer A-209R linked to a second Marantz PMD670 digital recorder (bandwidth: 20 Hz—20 kHz ± 1dB).

### Behavioural analyses

The videos of the birds’ behavioural responses were coded with a time-precision of one-tenth of a second with Solomon Coder Beta 15.01.15 (Copyright András Péter, http://solomoncoder.com). The birds’ behaviours was analysed separately for each of the three phases of the experiment (i.e. before, during, after). The following variables were coded: activity (the number of changes from one zone to another), number of calls emitted by the subject, time spent near the loudspeaker (designated as zone 1), and time spent with an erected crest (position 1, see S1 Fig). In particular, locomotion (movements from one zone to another), and the avoidance of the zone closest to the loudspeaker, were used to assess the emotional arousal of the birds and their motivation to flight. Vocalizations, on the other hand, are known to reflect emotional states in humans and non-human mammals [10, 68]. Consequently, the frequency of alarm calls (number of calls per experimental time unit) emitted in response to the distress calls of the emitter is used as proxy for the emotional state of the subjects [69]. Regarding the crest position, cockatiels are famous for their crest of feathers on top of their head, they can move when they are stressed or attentive. More generally, birds are known to puff their feathers to express emotional arousal in several contexts, such as during agonistic interactions or stressful events. For example, jackdaws exhibit a “bill-down” posture, which is a threat posture characterized by an erect body position combined with fluffed head and body feathers [70, 71]. Accordingly, we scored the duration, in which the crest was in the most erected position (position 1), which is related to a high level of stress and/or attention (see S1 Fig).

### Statistical analyses

Our first objective was to test whether the playback stimuli elicited behavioural reactions (i.e. higher frequency of behaviours during playback than before and after stimulation). We ran a generalized linear mixed model (GLMM) for each of our four response variables: activity (changes between zones), number of calls emitted, time spent near the loudspeaker (zone 1), and time spent with an erected crest (position 1). Models included the phase (before, during and after the playback stimuli) as fixed effect. Models also included individual identity, day of testing and the interaction between day of testing and order of stimuli as random effects.

The second objective was to investigate whether birds reacted more to distress calls than to white noise, and whether they responded more strongly to partner calls than to non-partner calls during and after the playback stimuli. For this, we ran two sets of GLMMs only using the data from the “during” or the “after” phases. The models included the type of playback stimulus (partner, non-partner, white noise) and the sex of the subject as fixed effects. Interactions could not be tested because the models did not converge. The models included individual identity, day of testing, and the interaction between day of testing and order of stimuli as random effects.

Finally, we created another set of GLMMs to investigate the birds’ behavioural responses before the stimuli (“before” phase) to ensure they were calm before playbacks. This set of GLMMs was identical to the second and third sets.

The ‘activity’ and ‘number of calls’ variables followed a Poisson distribution, while the variables ‘time spent near the loudspeaker’ and ‘duration of an erected crest (position 1)’ were binomially distributed (see S1 File); hence, we transformed them into binary variables by categorizing data as more or less than half the duration of a phase (i.e. 15 seconds) and used a binomial distribution. The three levels of each factor (i.e. “the phase” and “the type of playback stimulus” variables) were compared to each other by computing post-hoc Tukey tests with the “emmeans” package in R [72]. We checked for normality of random effects for all models and for overdispersion for Poisson distributed models. When the models were overdispersed, we corrected the standard errors by multiplying them by the square root of the dispersion parameter φ [73]. Corrected p-values were then computed using the new standard errors. All the results in the results section are given as a mean +/- SEM.

All statistical analyses were performed with R [74] using the LME4 R packages of Bates et al. (2015) [75].

## Results

### 1. Effect of the phase (“before”, “during” and “after” the playback stimulus) on the birds’ behaviour

**Fig 4:**
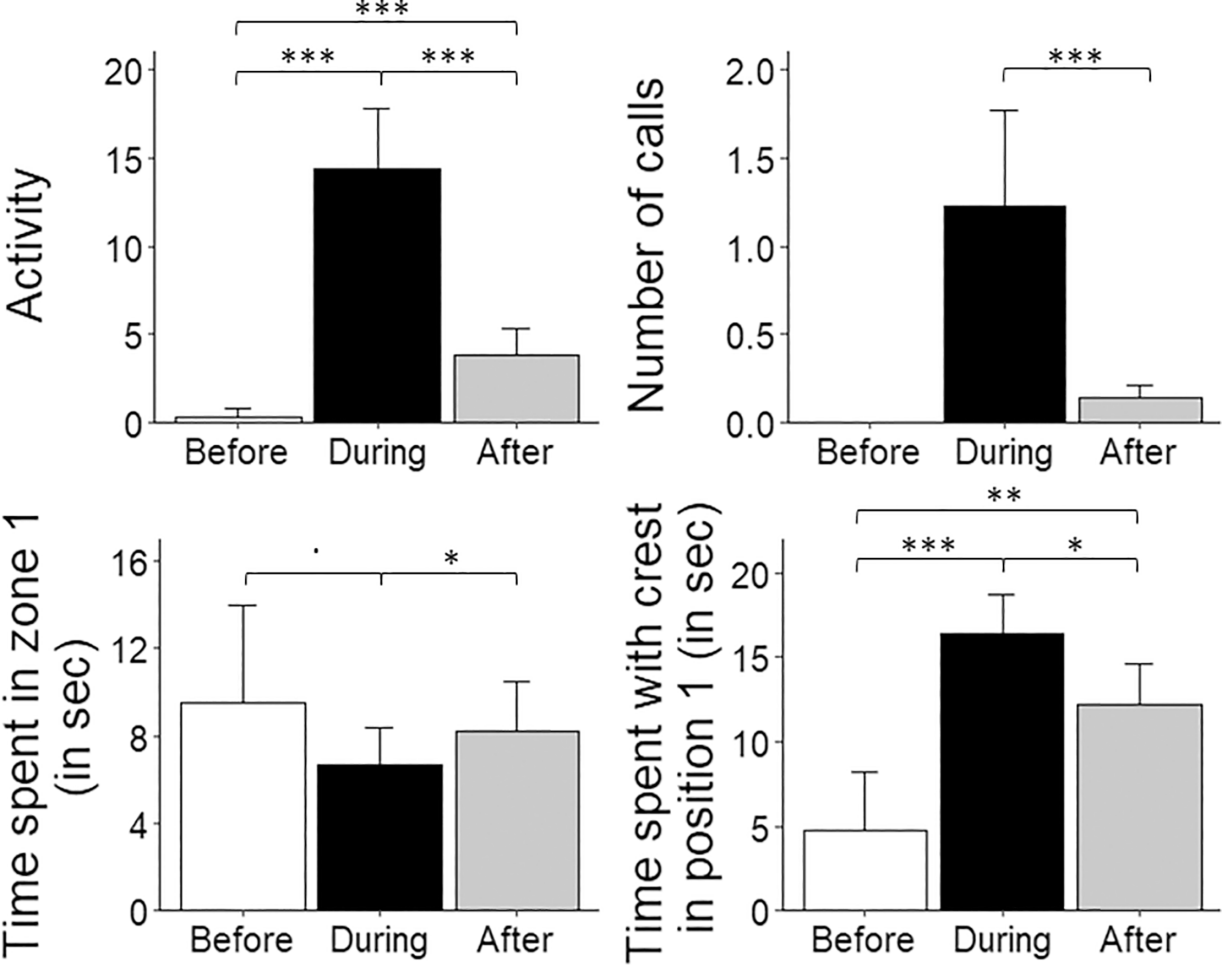
**Mean ± SE. of (A) activity (i.e. the number of zone changes), (B) number of calls emitted by the subjects, (C) the time the subjects spent in zone 1 (i.e. near the loudspeaker) and (D) the time the subjects spent with crest in position 1 (i.e. erected) in the phases before, during and after the playback stimuli.** Although the “time spent near the loudspeaker” and “time spent with crest in position 1” were analysed as binomial variables (see text), they are displayed here as continuous variables for illustrative purposes. Statistical differences between conditions (before, during, after) are given (***: *p* < 0.001; **: *p* < 0.01; *: *p* < 0.05;.: *p* = 0.07).

First, regarding the birds’ activity (number of zone changes), we found that the cockatiels were more active during the playback stimuli than before (during: 14.33 ± 1.74; before: 0.31 ± 0.25; *p* < 0.0001) and after (3.84 ± 0.78, *p* < 0.0001, φ = 2.49). Birds were also more active after the playback presentation than before (*p* < 0.0001; Fig 4A). Second, the phase of the experiment had a significant effect on the number of calls the subjects produced. Indeed, no calls were emitted before the playback stimuli and there were more calls emitted during the playback stimuli than after stimulation *(*during: 1.22 ± 0.28; after: 0.14 ± 0.04, *z* = -6.82, *p* < 0.0001, φ = 0.97; Fig 4B). Third, we observed a significant effect of phase on the time spent in zone 1. Birds spent less time in zone 1 during the playback than after (during: 0.14 ± 0.03; after: 0.27 ± 0.04, *p* = 0.03) and before (during: 0.31 ± 0.08, *p* = 0.07), although the difference between during and before playback was marginally significant. There was no difference between before and after phases (*p* = 0.90, Fig 4C). Fourth, the cockatiels exhibited an erected crest (position 1) more during presentation of the playback stimuli than before (during: 0.54 ± 0.05; before: 0.14 ± 0.06; *p* < 0.0001) or after the playback (0.41± 0.05, *p* = 0.007). An erected crest was also observed more frequently following the playback stimuli than before stimulation (*p* < 0.001; Fig 4D).

### 2. Behavioural responses to the different playback stimuli (partner, non-partner, white noise)

#### 2.1. Before playback stimuli

Birds appeared to be very calm before playback. There was no activity before playback of a partner’s call and only one female moved from a zone to another before playback of a white noise and a non-partner’s call. Moreover, no call was emitted by tested birds. There was no significant effect of the nature of the playback on time spent in zone 1 before playback (partner: 0.33 ± 0.14; non-partner: 0.25 ± 0.13; white noise: 0.33 ± 0.14; partner-white noise: *p* = 1.00; partner-non-partner: *p* = 0.88; non-partner-white noise: *p* = 0.88). Sex did not affect time spent in zone 1 (females: 0.38 ± 0.12; males: 0.22 ± 0.10; *p* = 0.30). Prior to playback of the distress calls or white noise, there was no difference in crest position. The cockatiels did not exhibit an erected crest (position 1) more often before being confronted with a partner’s distress call compared to before hearing a non-partner’s distress call (partner: 0.17 ± 0.11; non-partner: 0.08 ± 0.08; *p* = 0.21) or compared to before white noise (0.17 ± 0.11; *p* = 1.00). There was no significant difference in crest position before non-partner’s distress calls and before white noise (*p* = 0.21). As no males exhibited an erected crest more than 15 seconds, the effect of sex was not testable (females: 0.28 ± 0.11).

#### 2.2. During playback stimuli

**Fig 5:**
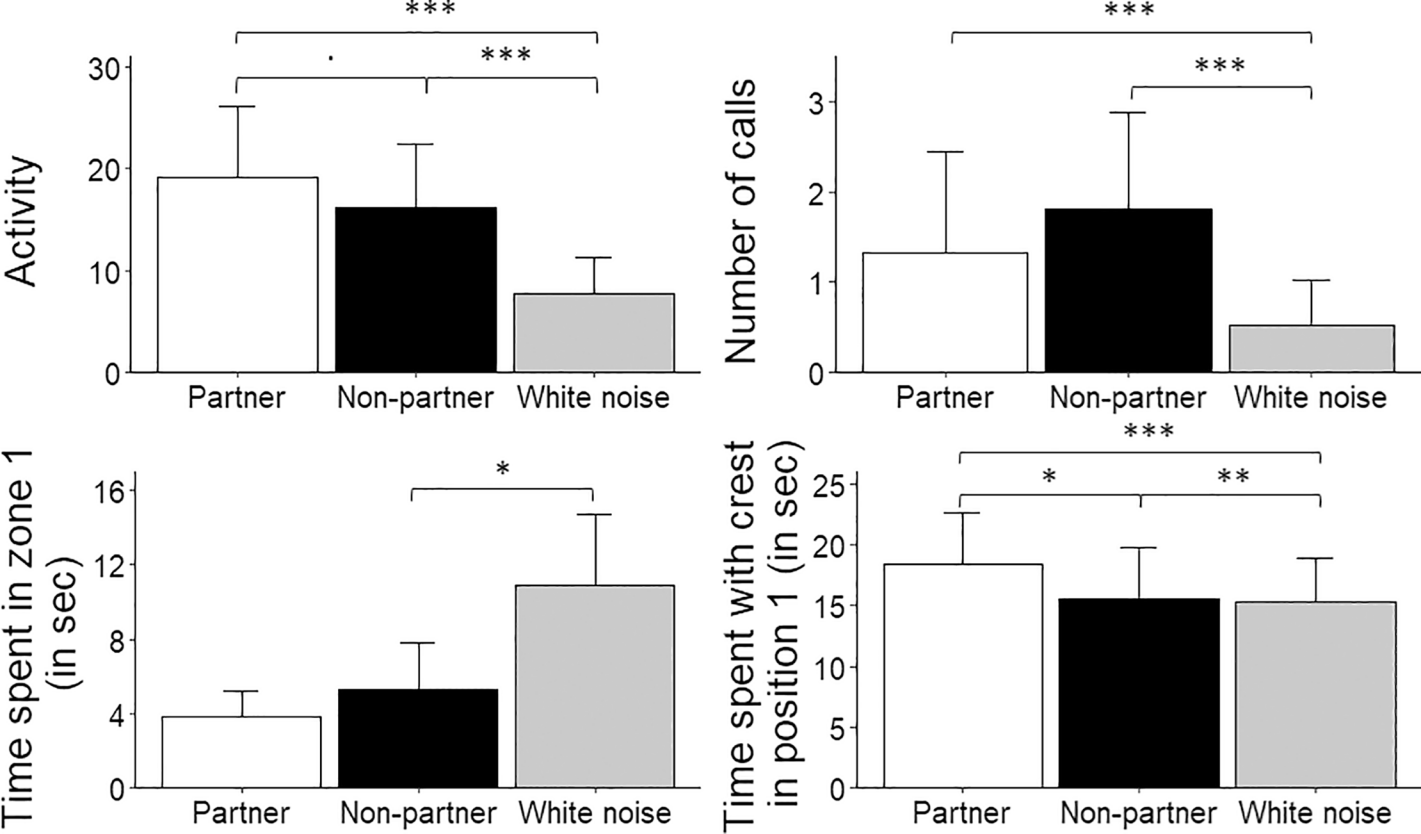
**Mean ± SE. of (A) activity (i.e. the number of zone changes), (B) number of calls emitted by the subjects, (C) the time the subjects spent in zone 1 (i.e. near the loudspeaker) and (D) the time the subjects spent with crest in position 1 (i.e. erected) during the playback of white noise, partner’s and non-partners’ distress calls.** Although the “time spent near the loudspeaker” and “time spent with crest in position 1” were analysed as binomial variables (see text for details), they are displayed here as a continuous variable for illustrative purposes. Regarding the time spent in zone 1, the differences between “partner” and other type of calls were not tested because no birds spent more than 15 seconds in zone 1 during the playback of partners’ distress calls (see text for details). Statistical differences between type of calls are given (***: *p* < 0.001; **: *p* < 0.01; *: *p* < 0.05;.: *p* = 0.07).

When looking only at the behaviours occurring during the playback stimuli, cockatiels were more active when confronted with a partner’s distress call than with white noise (partner: 19.17 ± 3.52; white noise: 7.69 ± 1.81; *p* < 0.0001, φ = 2.18) or a non-partner’s call (16.14 ± 3.19; *p* = 0.07), although this last difference was marginally significant. Subjects were also more active during non-partner’s distress calls than during white noise (*p* < 0.0001, Fig 5A). There was no effect of sex on activity (females: 10.00 ± 2.49; males: 18.66 ± 2.32; *p* = 0.09).

More calls were emitted during the playback of distress calls than during playback of artificial white noise (partner: 1.33 ± 0.57; non-partner: 1.80 ± 0.55; white noise: 0.53 ± 0.25; partner–white noise: *p* < 0.0001; non-partner–white noise: *p =* 0.0001; φ = 0.92). However, no significant difference was found in the number of calls emitted when a partner’s or a non-partner’s distress call was played back (*p* = 0.37, Fig 5B). There was no effect of sex on number of calls (females: 1.46 ± 0.48; males: 0.98 ± 0.30; *p* = 0.39).

Moreover, no bird spent more than 15 seconds in zone 1 near the loudspeaker during the playback of partners’ distress calls and they spent more time in zone 1 during the playback of white noise than during non-partners’ distress calls (non-partner: 0.11 ± 0.05; white noise: 0.31 ± 0.08; *p* = 0.03; Fig 5C). There was no sex effect on time spent near the loudspeaker (females: 0.15 ± 0.05; males: 0.13 ± 0.05).

Finally, birds displayed an erected crest (position 1) more often while listening to a partner’s distress call than to that of non-partner’s distress call (partner: 0.61 ± 0.08; non-partner: 0.53 ± 0.08; *p* = 0.05) or white noise (0.47 ± 0.08; *p* < 0.0001). Birds also displayed the crest in position 1 more often when hearing non-partners’ distress calls than artificial white noise (*p* = 0.002; Fig 5D). Females did the crest 1 position more often than males during playbacks (females: 0.63 ± 0.07; males: 0.44 ± 0.07; p < 0.0001).

#### 2.3. After playback stimuli

The cockatiels continued to be more active after playbacks of distress calls than after playbacks of white noise (partner: 4.69 ± 1.54; non-partner: 5.42 ± 1.52; white noise: 1.42 ± 0.79; partner–white noise: *p* < 0.0001; non-partner–white noise: *p* < 0.0001; φ = 2.04). However, no difference in activity was found between the phases after playbacks of partners’ or non-partners’ distress calls (*p* = 0.99). There was no sex effect on activity after playbacks (females: 2.15 ± 0.82; males: 5.54 ± 1.29; *p* = 0.09).

Similarly, no difference was found in the number of calls emitted after playback of a partner’s or a non-partner’s distress calls (partner: 0.22 ± 0.09; non-partner: 0.19 ± 0.08; *p* = 0.59, φ = 1.08). As no calls were emitted after playback of white noise, it was not possible to test for differences between the number of calls emitted after playbacks of distress calls and white noise. There was no sex effect on the number of calls after playbacks (females: 0.07 ± 0.04; males: 0.20 ± 0.07; *p* = 0.36).

The type of playback stimuli had an effect on the birds’ presence near the loudspeaker. They spent less time in zone 1 after playback of distress calls than after playback of white noise (partner: 0.17 ± 0.06; non-partner: 0.19 ± 0.07; white noise: 0.44 ± 0.08; partner–white noise: *p* = 0.02; non-partner–white noise: *p* = 0.04). No significant difference was found in the time spent near the loudspeaker after the playback of partner’s and non-partner’s distress calls (*p* = 0.94). There was no sex effect on the time spent in zone 1 after playbacks (females: 0.30 ± 0.06; males: 0.29 ± 0.06; *p* = 0.74).

Finally, there was no difference in time spent with the crest in position 1 after playback of partner’s distress calls, non-partner’s distress calls or white noise (partner: 0.44 ± 0.08; non-partner: 0.42 ± 0.08; white noise: 0.36 ± 0.08; partner–white noise: *p* = 0.49; non-partner–white noise: *p* = 0.71; partner—non-partner: *p* = 0.92). There was no difference between females and males for the time spent with the crest in position 1 (females: 0.57 ± 0.07; males: 0.24 ± 0.06; *p* = 0.09).

### 3. Individual variation

We observed individual differences between birds, as indicated by the high variance observed for the identity random effect during playback stimulation (activity: 1.69; number of calls: 4.17; zone 1: 1.94; crest 1, variance = 16.99) and after the playback phase (activity: 3.32; number of calls: 5.37; zone 1: 2.09; crest 1: 9.09).

## Discussion

Our results reveal that cockatiels reacted more intensely (as expressed by the activity of the bird, the number of calls, and the time spent near the loudspeaker and with an erected crest) to distress calls of conspecifics than to control noise. Furthermore, affiliation between birds affected their reaction to the distress calls, as the birds responded more strongly (e.g. stress-related behaviours: high activity, avoidance of the loudspeaker and erected crest) to partners’ distress calls than to non-partners’ distress calls. These results indicate that birds’ reaction to a conspecific’s distress call depends on the affiliation they shared with the emitter.

First, it is important to emphasize that hardly any such stress-induced behaviours were observed before the first playback stimulation (i.e. “before” phase), suggesting that social isolation in itself or the handling at the beginning of the experiment did not elicit any observable arousal. Before the first playback stimulus, the birds were not very active, did not emit a single call and exhibited a crest in position 3 (see S1 Fig). They typically sat calmly on the floor and appeared sleepy at the end of the “before” phase preceding the playback. It may be assumed that this sleepiness could have been a consequence of the stress they felt before the beginning of the experiment when handled, as it has been observed in budgerigars (*Melopsittacus undulatus*) [76]. Stress-related behaviours were mostly exhibited during playback stimulations and, to a lesser extent, after these playbacks. This means that audio stimulations elicited a very quick, but prolonged, behavioural response in the receivers.

Our most important result is that cockatiels’ reaction to their conspecific distress calls was influenced by the degree of affiliation they shared. Cockatiels spent less time near the loudspeaker, spent more time with an erected crest and tended to be more active when hearing distress calls emitted by a conspecific with which they maintained a strong affiliative bond in comparison to distress calls emitted by a familiar bird housed in the same aviary but without affiliation to the subject. These results suggest that distress calls convey emotional information in cockatiels and that affiliation further modulates emotional response to the vocalization. Similar results were obtained in females geladas baboons (*Theropithecus gelada*) in which the contagiousness of yawning correlated with affiliation and the level of grooming contact between partners [77]. However, this is the first study, to our knowledge, showing that affiliation modulates emotional response in birds. Ravens have been shown to respond differently to the playbacks of conspecifics’ calls depending on the valence of their relationships with the emitter of the calls [78]. However, this study focused on the ravens’ memory for conspecific calls rather than emotional responses per se.

It is important to underline that we used a control sound (white noise) to ensure the birds’ reaction to distress calls was driven by the emotional valence of the stimuli rather than by the sound itself. The control sound did not elicit a similarly strong behavioural reaction during and following the stimulus presentation in comparison to the distress calls. Indeed, birds were less active, emitted less calls, spent more time near the loudspeaker and less time with their crest erected during and after (except for the crest position) the presentation of the control sound in comparison to distress calls. Similar results have been obtained in domestic dogs (*Canis familiaris*) which exhibited more stress-related behaviours when exposed to conspecifics’ whines than when hearing control stimuli [20]. Regarding activity, similar results have been found in another bird species, the Indian mynahs (*Acridotheres tristis*) which increased its flight and walking rates when exposed to a taxidermic model hawk and the associated distress calls from conspecifics, as compared to blank controls trials [79]. This increase in locomotor activity may reflect their motivation to escape because distress calls are known to elicit strong aversive reactions and immediate flight responses e.g. used in airports to scare away birds from aircrafts [80]. However, some bird and bat species’ distress calls alert conspecifics while also triggering their mobbing behaviour, thus attracting conspecifics [81–83]. In the present study, the subjects significantly avoided the loudspeaker during the playback of distress calls, but not white noise, which suggests an aversive reaction to the distress calls, potentially resulting in flight behaviour rather than mobbing behaviour.

One of the limits in this study may be the use of white noise as a control instead of a social stimulus. We thought about using different type of control sounds like calls from cockatiels recorded in their aviary without the birds being stressed. But these controls would have been problematic due to several possible uncontrollable confounding factors. By using the white noise, our goal was to use an artificial noise, not emitted by any living bird and with no emotional meaning at all. Even if we used a call of a cockatiel outside any stressful context, we doubted that this sound would be perceived as “neutral” by the receiver. Indeed, even social vocalizations emitted in the aviary during a peaceful moment likely have a meaning that is unknown to us. Consequently, we kept the white noise as a neutral control which has been used in a previous study similar to ours [19].

To summarize, cockatiels reacted more strongly to conspecific’s distress calls compared to a control sound. Interestingly, they reacted differently depending on the identity of the emitter and the degree of affiliation between them (e.g. activity, avoidance of the loudspeaker and crest erection). These results suggest that distress calls convey emotional information in cockatiels and that affiliation further modulates emotional contagion of the vocalization. Additionally, we could establish the position of the crest feathers as a meaningful indicator for attentiveness and emotional arousal in cockatiels. Although we exclusively focused on negatively valued calls, our results suggest that it may be promising to further assess emotional contagion and empathy in psittacids.

## Supporting information

S1 Data Supporting data. (XLSX)

S1 Table Randomization of conditions depending on the subjects and the days of testing. P: Partner, NP: Non-partner, WN: White Noise.
(PDF)

S1 Fig Schematic representation of the crest positions. Crest 1 position is characteristic of a stressed or attentive bird, with clearly separated feathers. The second position is intermediate and is characteristic of a middle stressed or attentive bird. The Crest 3 position is observed in entirely relaxed birds e.g. while resting.
(DOCX)

S1 File Histograms of activity, number of calls emitted, time spent in zone 1 and time spent in crest position 1 during a phase. Results from all type of phases (before, during, after) and all type of calls (partner, non-partner, white noise) are pooled.
(PDF)
