# Supplementary material for: Emotional responses to conspecific distress calls are modulated by affiliation in cockatiels (Nymphicus hollandicus)
Source: PLoS One. 2018 Oct 9;13(10):e0205314. doi: 10.1371/journal.pone.0205314 (PMC6177178; doi:10.1371/journal.pone.0205314)
Supplement: S1 Table — P: Partner, NP: Non-partner, WN: White Noise. (PDF) [file pone.0205314.s002.pdf]

Table S1. Randomization of conditions depending on the subjects and the days of testing. P: Partner, NP: Non-partner, WN: White Noise.

Days of testing / Subjects	Day 1	Day 2	Day 3
**Callisto**	NP	WN	P
**Hermes**	WN	P	NP
**Nephtys**	P	NP	WN
**Seth**	WN	P	NP
**Sita**	P	NP	WN
**Viviane**	WN	P	NP
**Wala**	NP	WN	P
**Loki**	NP	WN	P
**Odin**	P	NP	WN
**Skadi**	WN	P	NP
