# Supplementary material for: Emotional responses to conspecific distress calls are modulated by affiliation in cockatiels (Nymphicus hollandicus)
Source: PLoS One. 2018 Oct 9;13(10):e0205314. doi: 10.1371/journal.pone.0205314 (PMC6177178; doi:10.1371/journal.pone.0205314)
Supplement: S1 Fig — Crest 1 position is characteristic of a stressed or attentive bird, with clearly separated feathers. The second position is intermediate and is characteristic of a middle stressed or attentive bird. The Crest 3 position is observed in entirely relaxed birds e.g. while resting. (DOCX) [file pone.0205314.s003.docx]

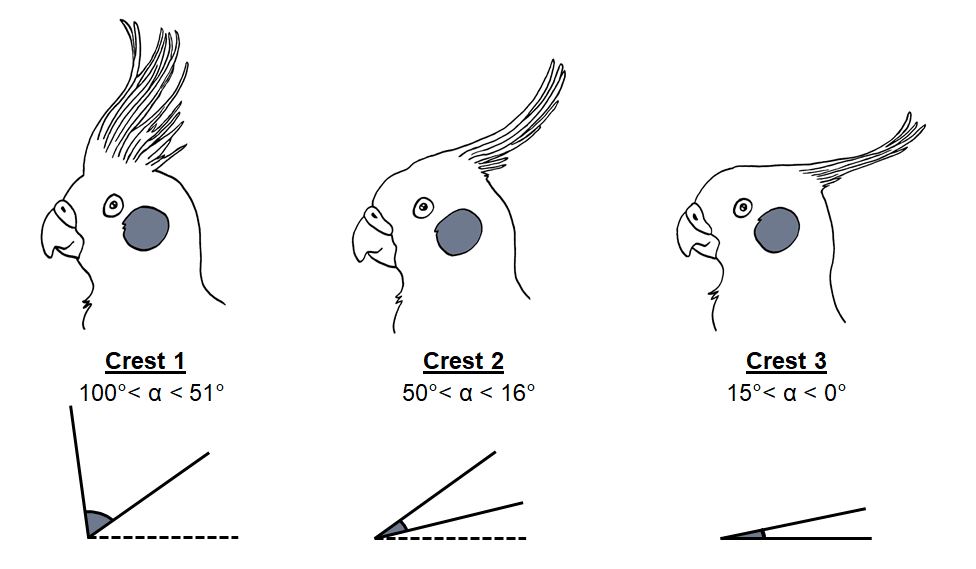


**Figure S1. Schematic representation of the crest positions**. Crest 1 position is characteristic of a stressed or attentive bird, with clearly separated feathers. The second position is intermediate and is characteristic of a middle stressed or attentive bird. The Crest 3 position is observed in entirely relaxed birds e.g. while resting.
