# Supplementary material for: Emotional responses to conspecific distress calls are modulated by affiliation in cockatiels (Nymphicus hollandicus)
Source: PLoS One. 2018 Oct 9;13(10):e0205314. doi: 10.1371/journal.pone.0205314 (PMC6177178; doi:10.1371/journal.pone.0205314)
Supplement: S1 File — Results from all type of phases (before, during, after) and all type of calls (partner, non-partner, white noise) are pooled. (PDF) [file pone.0205314.s004.pdf]

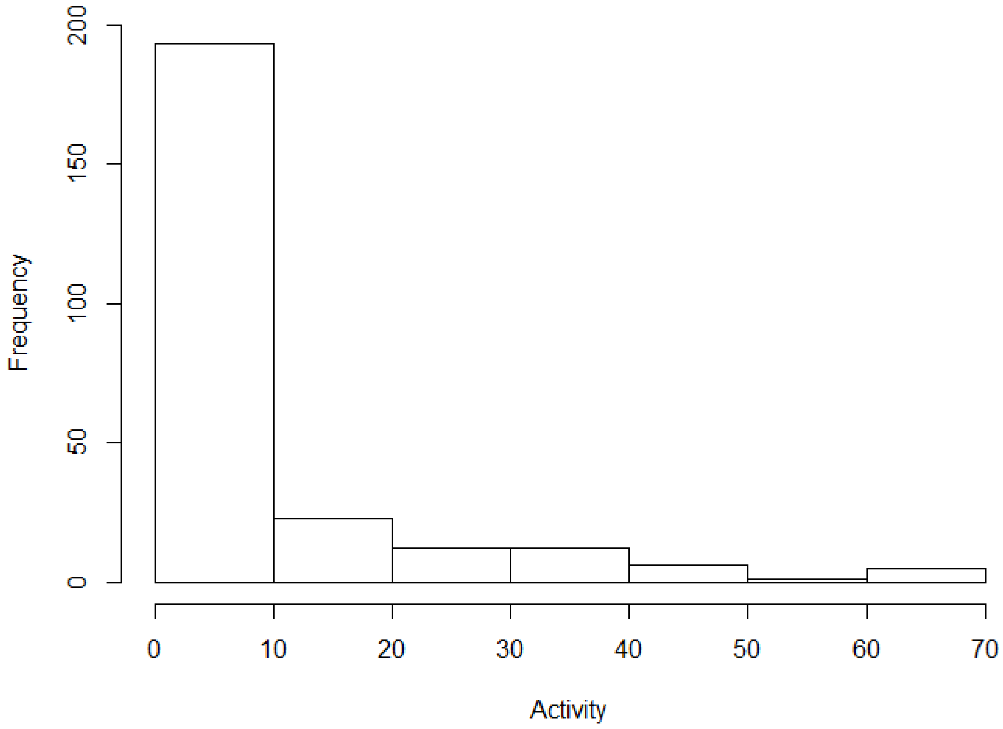

Figure S2. Histogram of activity, i.e. number of zone changes, during a phase. Results from all type of phases (before, during, after) and all type of calls (partner, non-partner, white noise) are pooled.

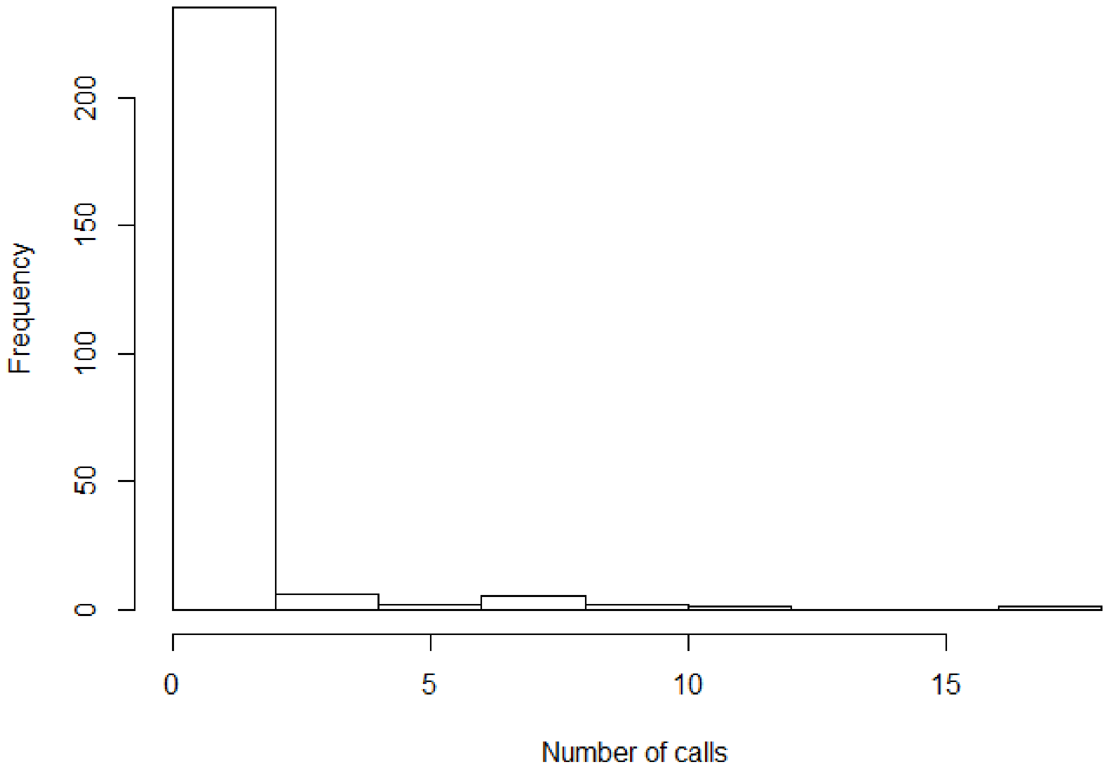

Figure S3. Histogram of number of calls emitted during a phase. Results from all type of phases (before, during, after) and all type of calls (partner, non-partner, white noise) are pooled.

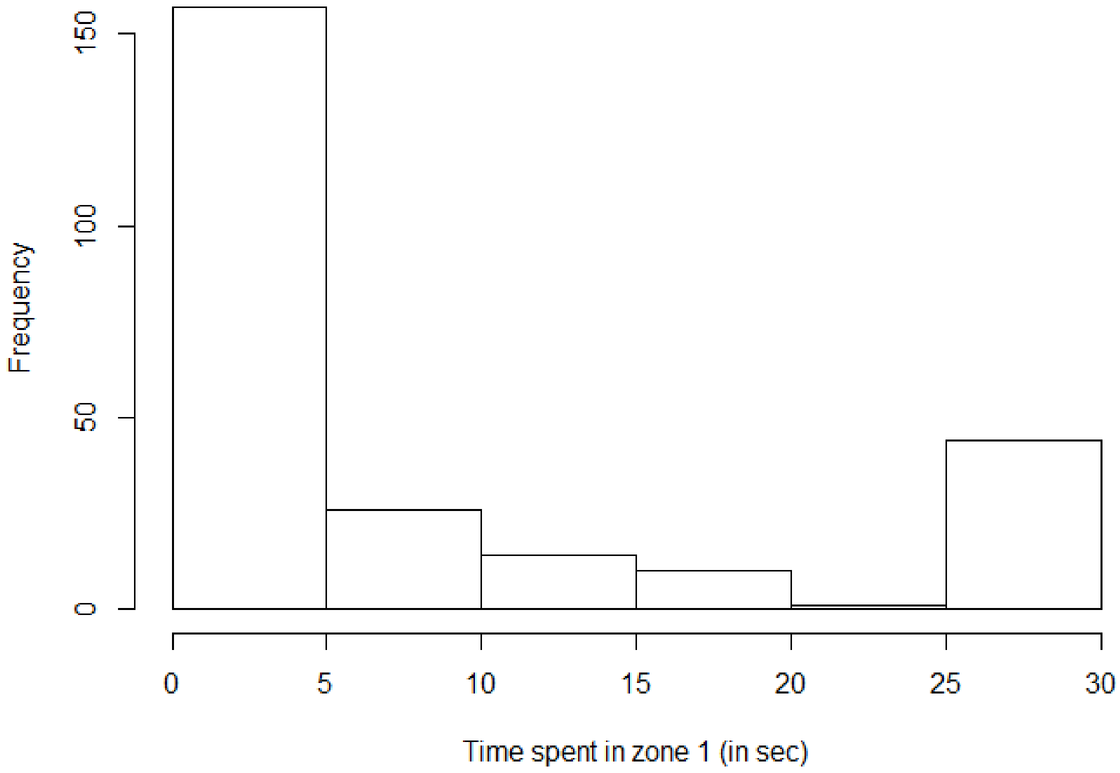

Figure S4. Histogram of time spent in zone 1, i.e. near the loudspeaker, during a phase (max. 30 seconds). Results from all type of phases (before, during, after) and all type of calls (partner, non-partner, white noise) are pooled.

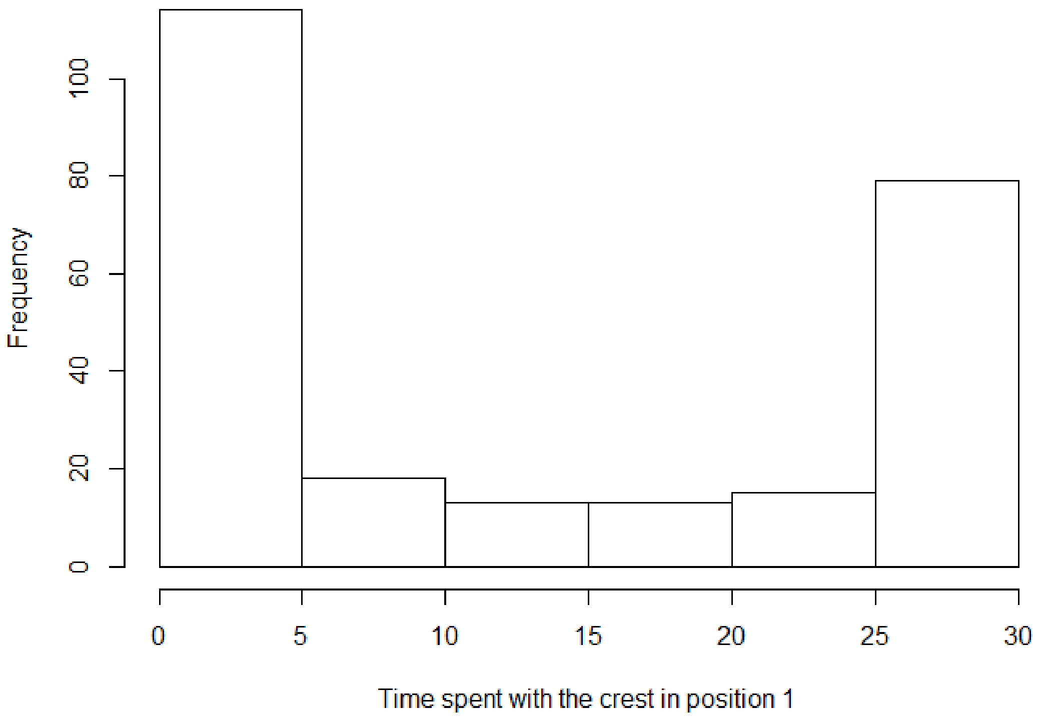

Figure S5. Histogram of time spent with crest in position 1, i.e. erected, during a phase (max. 30 seconds). Results from all type of phases (before, during, after) and all type of calls (partner, non-partner, white noise) are pooled.
